# Embleporicin: A Novel Class I Lanthipeptide from the Actinobacteria *Embleya* sp. NF3

**DOI:** 10.3390/antibiotics13121179

**Published:** 2024-12-05

**Authors:** Dora Onely Roblero-Mejía, Carlos García-Ausencio, Romina Rodríguez-Sanoja, Fernando Guzmán-Chávez, Sergio Sánchez

**Affiliations:** 1 Instituto de Investigaciones Biomédicas, Universidad Nacional Autónoma de México (UNAM), Mexico City 04510, Mexico; onely.roblero95@gmail.com (D.O.R.-M.); carlos_adgar@iibiomedicas.unam.mx (C.G.-A.); romina@biomedicas.unam.mx (R.R.-S.); 2 Departamento de Alimentos y Biotecnología, Facultad de Química, Universidad Nacional Autónoma de México (UNAM), Mexico City 04510, Mexico

**Keywords:** actinobacterium *Embleya* sp. NF3, genome mining, lanthipeptides, antimicrobials, cell-free expression

## Abstract

Genome mining has emerged as a revolutionary tool for discovering new ribosomally synthesized and post-translationally modified peptides (RiPPs) in various genomes. Recently, these approaches have been used to detect and explore unique environments as sources of RiPP-producing microorganisms, particularly focusing on endophytic microorganisms found in medicinal plants. Some endophytic actinobacteria, especially strains of *Streptomyces*, are notable examples of peptide producers, as specific biosynthetic clusters encode them. To uncover the genetic potential of these organisms, we analyzed the genome of the endophytic actinobacterium *Embleya* sp. NF3 using genome mining and bioinformatics tools. Our analysis led to the identification of a putative class I lanthipeptide. We cloned the core biosynthetic genes of this putative lanthipeptide, named embleporicin, and expressed them in vitro using a cell-free protein system (CFPS). The resulting product demonstrated antimicrobial activity against *Micrococcus luteus* ATCC 9341. This represents the first RiPP reported in the genus *Embleya* and the first actinobacterial lanthipeptide produced through cell-free technology.

## 1. Introduction

Lanthipeptides, the largest sub-family of ribosomally synthesized and post-translationally modified peptides (RiPPs), are characterized by lanthionine (Lan) or methyllanthionine (MeLan) structures [1,2]. These peptides are formed by the dehydration of Ser/Thr residues to dehydroalanine (Dha) and dehydrobutyrine (Dhb) residues, respectively, followed by the Michael-type addition of sulfhydryl groups of Cys residues to dehydroamino acids [3,4]. Genes involved in lanthipeptide biosynthesis are typically encoded in biosynthetic gene clusters (BGCs) [5], and they are categorized into five classes based on biosynthetic enzymes [6,7]. Regarding class I, the precursor peptide (known as LanA) is modified by a dehydratase enzyme (LanB), which is responsible for the activation and elimination of the chain hydroxyl groups of Ser and Thr residues [8] and subsequently cyclized by cyclase enzyme (LanC), which is responsible for thioether formation [9]. The nisin from *Lactococcus lactis* subsp. *lactis* represents the class I lanthipeptide that is more widely studied [10,11]. Lanthipeptides can exhibit various biological activities, including antimicrobial activities [2,12]. Some examples include planosporicin [13], cebulatin [14], microbisporicin [15], and lexapeptide [16]. These are commonly referred to as lantibiotics, which even have activity against multi-resistant pathogens [2]. As a result, discovering new lantibiotics is a promising strategy to combat antimicrobial resistance (AMR) [6].

The development of genomic mining and the increasing availability of bacterial genome data have revealed an unexplored source of lanthipeptides in microorganisms, mainly in Gram-positive bacteria [17,18,19] and in some cases on Gram-negative [20] and archaea genomes [21]. The processing and analysis of such information has recently led to identifying BGCs lanthipeptide in Actinobacteria genomes and has revealed lanthipeptides that exhibit unique characteristics often associated with increased biological activity [7]. In this context, antiSMASH (Antibiotics and Secondary Metabolite Analysis Shell) has been a valuable tool for predicting BGCs of lanthipeptides and other secondary metabolites in microbial genomes [22,23]. Other genome mining tools available for lanthipeptide BGCs are BAGEL4, RiPPMiner, RODEO2, and PRISM4 [24]. However, many of these BGCs identified in microbial genomes are often silent or poorly expressed in their native strains. Additionally, they may be found in unculturable strains within laboratory or metagenomic samples. Furthermore, we currently lack the understanding as well as the necessary tools to activate the expression of their BGCs under laboratory conditions [25,26,27].

One way to improve the yield of lanthipeptide production is to choose DNA-based bioproduction methods. Some lanthipeptides have been produced by inserting strong promoters into the genome in the original bacteria to activate the silent BGCs or reconstructing their biosynthetic pathways and expressing them in heterologous hosts [6,28]. However, a limitation of this last method is heterologous hosts, such as *Escherichia coli*, pose unavoidable challenges: intracellular toxicity, frequent formation of inclusion bodies, and time and labor-intensive [29,30]. Cell-free protein synthesis (CFPS) offers a promising solution to these challenges [31], becoming a powerful tool for the synthesis of lanthipeptides. Likewise, due to the antimicrobial properties of lantibiotics, microbes need to produce transporters and immunity proteins within their BGCs to ensure effective protection. In contrast, CFPS systems eliminate the need to produce such proteins because the biosynthesis of peptides is independent of cell growth. This approach mitigates potential cellular toxicity and facilitates the rapid, small-scale production of several peptides/proteins from linear DNA in parallel [32,33,34].

In our research group, seeking new compounds of microbial origin to manage AMR has led us to explore non-model microorganisms. *Embleya* sp. NF3 (previously known as *Streptomyces scabrisporus* NF3) is an actinomycete isolated and selected from *Amphipterygium adstringens*, a medicinal plant found in some regions of México [35]. The preliminary genomic sequence of 10,760,685 bp comprises eight scaffolds and 10,492 coding sequences (CDS), with 5691 predicted genes and 4729 hypothetical proteins [36]. This study aimed to conduct an in silico analysis of a cluster that encodes a class I lanthipeptide derived from the genome of *Embleya* sp. NF3. We named this peptide “embleporicin”, a name that reflects its origin from the actinomycete (“emble”) and its classification among class I lanthipeptides found in actinobacterial genomes, such as planosporicin and microbisporicin (“poricin”). We expressed this peptide using cell-free protein synthesis (CFPS). We assessed its antimicrobial activity against *Micrococcus luteus* ATCC 9341, which serves as a model bacterium for evaluating the antimicrobial properties of lanthipeptides known for their effectiveness primarily against Gram-positive bacteria [31,37].

This research represents the first report of a class I lanthipeptide from an actinomycete biosynthetic gene cluster (BGC) successfully expressed using CFPS.

## 2. Results

### 2.1. Bioinformatical Analysis of a Putative Cluster of Class I Lanthipeptide

*Embleya* sp. NF3 is a strain known for producing the anticancer compound steffimycin B and its derivatives [35,38]. Bioinformatic analysis of this strain has revealed its significant potential as a producer of other new natural products, including non-ribosomal peptides, polyketide synthases, siderophores, and RiPPs such as lasso peptides, thiopeptides, and lanthipeptides encoded in its genome [39,40] (Figure S1). To date, no lanthipeptides have been reported in the genus *Embleya*. Therefore, we focused on a class I lanthipeptide cluster for bioinformatic characterization and heterologous expression.

**Figure 1:**
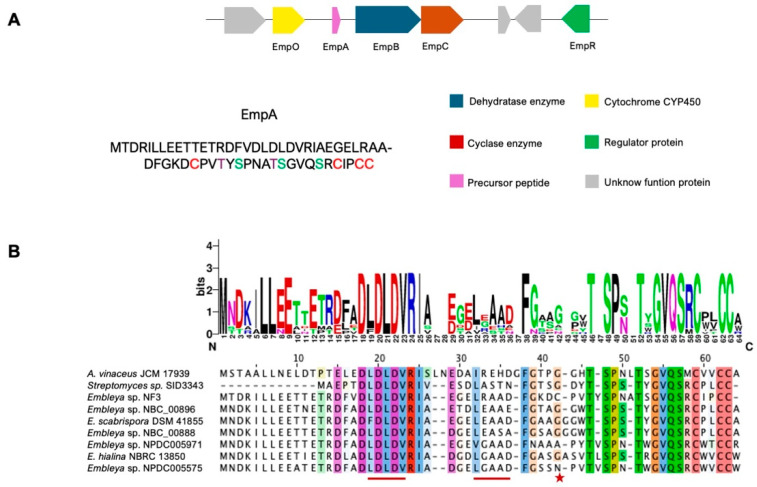
Schematic representation of embleporicin cluster and EmpA sequence. (**A**) Organization of the main genes of embleporicin cluster in *Embleya* sp. NF3 and (**B**) alignment of homologous sequences to embleporicin is marked with a red line for the conserved motifs and putative cutting sites. In contrast, the red star shows the additional cysteine residue of embleporicin. The accession number of alignment sequences is shown in Table S3.

The AntiSMASH v. 7.1.0 analysis identified three clusters associated with the production of class I lanthipeptides in the *Embleya* genome (Figure S2). One of these clusters (2.3) contains the precursor peptide EmpA, along with the main enzymes EmpB and EmpC, which are involved in the production of embleporicin. This gene cluster is predicted to consist of 18 genes within a 24,462-nucleotide region of genomic DNA. Among these genes are those responsible for the precursor peptide, core biosynthetic functions, additional biosynthetic enzymes, regulatory proteins, and other related genes. (Figure 1, Table S1).

The precursor peptide gene *empA* encodes a 59-residue peptide consisting of a 33-residue leader peptide followed by a predicted 26-residue core peptide. This core region contains three serines, two threonines, and four cysteines, which may allow the formation of a four-ring cyclized peptide. Moreover, the presence of the LDLD motif in the leader peptide suggests that embleporicin likely belongs to the class I lanthipeptide group [37,41].

A Blast-P search of the peptide sequence revealed alignments with some members of the SCO0268 family, a proposed group of lanthipeptides found in various *Streptomyces* strains [42]. The SCO0268 family was first identified in a genome mining study conducted in 2010 and is characterized by the PEAQxS motif [5,43]. Notably, embleporicin does not contain this motif in its sequence.

**Figure 2:**
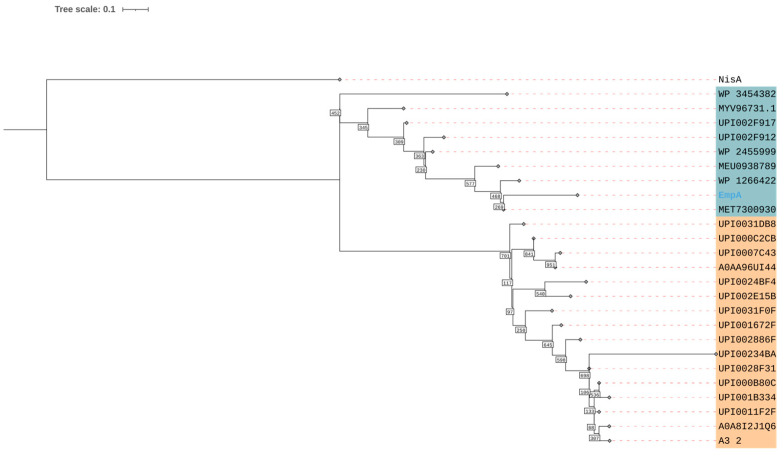
Phylogenetic tree of homologous proteins of embleporicin. In orange, SCO0268 members are indicated, while homolog peptides of embleporicin are shown in blue. The accession sequences are shown in Table S3.

To confirm whether embleporicin is part of this lanthipeptide family, we aligned its peptide sequence with that of the SCO0268 family and generated a phylogenetic tree (see Figure 1B and Figure 2).

The alignment indicates the absence of the motif PEAQxS. However, we observed that the C-terminal region of EmpA shares similarities with the core region sequences from SCO0268. Despite this similarity, the phylogenetic tree shows that embleporicin does not belong to the SCO0268 group. Nevertheless, EmpA retains a conserved motif found among various *Embleya* peptides, specifically a semi-conserved LDLD motif in the leader peptide and a GVQSxCxxCCx motif in the core peptide (Figure 1B). In contrast, embleporicin potentially possesses four rings instead of three, unlike the sequences in the SCO0268 family, according to the presence of cysteine residues in core peptides. These observations suggest that embleporicin has a unique structure among actinobacterial lanthipeptides.

The bioinformatic analysis of the protein sequences has revealed the putative functions of other biosynthetic genes involved in embleporicin production. The EmpB and EmpC enzymes are responsible for the post-translational modifications of EmpA, specifically dehydration and cyclization, respectively. The *empB* gene contains an open reading frame of 3201 nucleotides, corresponding to 1067 amino acid residues, and has a theoretical molecular mass of 118 kDa. In contrast, the *empC* gene consists of 1383 nucleotides, encodes a protein of 460 amino acids, and has a theoretical molecular mass of 48.9 kDa.

Both enzymes were aligned with other lanthionine synthetases to identify conserved motifs. The amino acid sequences of the post-translational modifying enzymes were compared with lantibiotic enzyme sequences from microbisporicin, planosporicin, and cebulantin producers, specifically from the genomes of *Microbipora corallina*, *Planomonospora alba*, and *Saccharopolyspora cebuensis*, respectively (Figure S3). The sequences of the nisin lantibiotic enzymes from *L. lactis* subsp. *lactis* were also considered.

The EmpB enzyme exhibited higher identity with actinobacterial lantibiotic dehydratases: PspB from planosporicin (32.1%), MibB from microbisporicin (30.2%), and CebB from cebulantin (26.2%). In contrast, a lower identity of 20.1% was observed with NisB, the nisin dehydratase. This finding aligns with the phylogenetic relationship to the genus *Embleya* [44]. Furthermore, in most instances, the conserved amino acid residues responsible for glutamylation and the scavenging of glutamate in the Ser/Thr residues of the precursor peptide, as reported for NisB [45], are not conserved (see arrows in Figure S3A). However, these residues are often identical to those in CebB, suggesting that these two lantibiotic dehydratases may employ different mechanisms for the glutamylation and scavenging processes. Similarly, the EmpC enzyme shares a lower identity with NisC (18.9%) and a higher identity with MibC (34.4%), as well as with PspB (32.2%) and CebB (25.5%). Importantly, the amino acid residues of the catalytic triad involved in zinc binding are conserved [46] (see arrows in Figure S3B). Additionally, structural models of the enzymes were constructed using AlphaFold to compare with the crystalized structures of other lantibiotic enzymes, MibB from *Microbispora* sp. 107891782 and NisC from *L. lactis* subsp. *lactis* (Figure S4A,B) [47].

Although the *emp* cluster does not contain homologous genes coding for the LanP enzyme, which is generally derived from subtilisin-like serine proteases, it has been observed that Actinobacterial lanthipeptide biosynthetic gene clusters typically lack a protease within the biosynthetic cluster itself. Instead, such enzymes may reside elsewhere in the genome [48,49].

Interestingly, an open reading frame (ORF) coding for a gamma-glutamyl gamma-aminobutyrate hydrolase was identified at 6852 base pairs from *empA*. This enzyme, part of the C26 family of proteases, is widely found in bacteria and animals and functions as an endopeptidase [50]. However, no members of this family have been reported as the primary protease involved in the processing of lanthipeptides to date [51].

To produce mature embleporicin, it is necessary to cleave the leader peptide proteolytically. Prediction algorithms indicate a potential recognition site at LRAA-DFGK. Notably, this site is missing proline at position-2 (Pro-2), which is conserved in some class I lanthipeptides [37,52]. This omission suggests that a non-specific protease may be responsible for the cleavage. Further research is needed to confirm this hypothesis.

Other genes within the *emp* cluster include a domain-containing protein with a DUF4259 domain and an amino acid-polyamine carbocation (APC) permease, which is characterized by its ability to transport a wide variety of solutes [53]. This permease is rarely found in other gene clusters of RiPPs, and it likely plays a role in the export of embleporicin. Additionally, a cytochrome P450 (CYP450) was detected upstream of *empA*. CYP450 enzymes can catalyze oxidations of precursor peptides and introduce various functional groups [41,54], such as the hydroxyl group found in microbisporicin. It is also possible that CYP450 may facilitate macrocyclization in other RiPP families [55,56]. According to the nomenclature for microbisporicin, EmpO shows 26.9% identity with CYP450 MibO (ADK32549.1), and its likely functions as an oxidant for certain amino acids, such as aspartate (Asp), in the embleporicin core peptide.

In addition, antiSMASH identified two regulatory proteins: a MarR family transcriptional regulator (located near the APC permease) and a TetR family transcriptional regulator. The latter, known as EmpR, is a homolog of TetR/AcrR family transcriptional repressors. These repressors are known to regulate processes such as antibiotic biosynthesis and multidrug resistance [57,58]. The AlphaFold model of EmpR confirms the presence of ten alpha-helices, a characteristic structure found in this family of regulators [59] (Figure S4C).

Recently, a new regulator, AcrR1, was described as a repressor of nisin production in *Lactococcus lactis* F44, specifically inhibiting the production of the component NisC [60]. Notably, EmpR has only 18.1% identity with AcrR1, likely because the similarity is restricted to the DNA-binding domain in the N-terminal region, while the remainder of the sequence is tailored for auto-dimerization and ligand binding [61,62].

Despite an extensive search for the recognition motif of TetR/AcrR regulators within the embleporicin cluster, we could not identify a consensus sequence. Additionally, a HU family DNA-binding protein is located near the *emp* cluster, suggesting that histone-like proteins are involved in DNA repair [63,64].

Interestingly, AntiSMASH identified other proteins involved in various enzymatic processes. These include oxide-reduction enzymes such as one aldehyde dehydrogenase, one acetoacetate decarboxylase, and one alcohol dehydrogenase, which could participate in the ability of the strain [40] to utilize other carbon sources in central metabolism. The embleporicin biosynthetic gene cluster (BGC) contains one glutamine synthetase, a gamma carbonic anhydrase family protein, and a flavohemoprotein. Surprisingly, no additional modification enzymes were found near the precursor gene (*empA*).

To produce the active version of embleporicin, we hypothesize that the expression of each enzyme in the biosynthetic core (EmpB, EmpC, and the precursor peptide EmpA) is essential [32,65]. Therefore, we propose cloning and expressing these genes to evaluate the biological activity of embleporicin.

### 2.2. Obtention of Embleporicin by CFPS and Antimicrobial Assay

The CFPS has been proven to be an invaluable tool for evaluating the bioactivity of microbial peptides more quickly and cost-effectively [34]. However, to our knowledge, no actinobacterial lanthipeptides have been produced using this technology, prompting us to apply CFPS to synthesize embleporicin.

**Figure 3:**
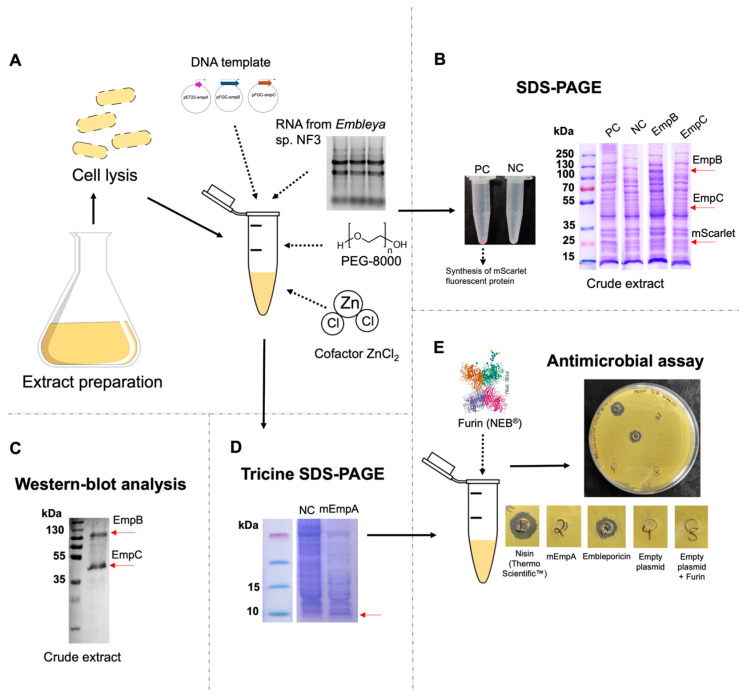
Reconstitution of embleporicin biosynthesis using the CFPS system. (**A**) Extract preparation and addition of other extract components for the synthesis of embleporicin. (**B**) SDS-PAGE electrophoresis of the crude extract to visualize the post-translational modifying enzymes (EmpB and EmpC). The red arrows indicate the expected size of the enzymes; PC means positive control, and NC means negative control. (**C**) Western-blot analysis of the post-translational modifying enzymes (EmpB and EmpC). (**D**) Tricine SDS-PAGE to visualize the modified peptide (mEmpA). The red arrow indicates the expected size of the mEmpA, and NC means negative control. (**E**) Addition of Furin to the in vitro reaction and an antimicrobial assay of the modified peptide (mEmpA) and the mature peptide (embleporicin). Nisin (Thermo Scientific, Waltham, MA, USA) was used as a positive control, and empty plasmids with and without the addition of Furin were used as negative controls.

Each of the constructions, pET22-*empA*, pFGC-*empB*, and pFGC-*empC*, were added individually to the concentration of 1.6 nM of each plasmid (5 nM in total) and incubated for 14 h. The expression of EmpB and EmpC enzymes was corroborated in western-blot analysis (Figure 3C) when we noted that EmpC enzyme was more visible than EmpB enzyme, which was not very visible (Figure S5). In both cases, the bands correspond to the biosynthetic core, around 120 kDa for EmpB and 51 kDa for EmpC (including the weight of the His-6X tag). Apart from that, the EmpA expression was observed in Tricine SDS PAGE (Figure 3D); despite carrying out western-blot analysis, this peptide was not visible in either assay.

To obtain mature embleporicin, we combined the three constructs into a single reaction microtube. We added total RNA from *Embleya* sp. NF3, along with ZnCl_2_, into the same reaction mixture. Notably, the reaction that excluded both ZnCl_2_ and RNA from *Embleya* sp. NF3 did not exhibit any antimicrobial activity. It has been observed that LanB enzymes specifically select tRNA-Glu from their host [66,67], which is why we needed to extract total RNA from *Embleya* sp. NF3. Additionally, ZnCl_2_ is potentially important as a cofactor for LanC enzymes. Zinc plays a vital role in the cyclase (EmpC), where it binds to the strictly conserved Cys–Cys–His catalytic triad near the C-terminal ends, facilitating the nucleophilic attack on dehydroamino acids [68,69].

Following this, we adhered to the protocols for nisin production [31] and added Furin protease after the incubation period with the templates pET22-*empA*, pFGC_*empB*, and pFGC_*empC* to digest the leader peptide of modified embleporicin. The samples were concentrated and plated on agar with *M. luteus* ATCC 9341. After 18 h, we observed a visible inhibition halo, indicating activity comparable to nisin at 90 UI. In contrast, when Furin was not included in the in vitro reaction containing the modified peptide (mEmpA), no antimicrobial activity was detected (Figure 3E). This emphasizes the critical role of the endoproteinase Furin in cleaving the leader peptide from mEmpA, reinforcing the notion that EmpA must be fully modified to exhibit biological activity. Previous studies have shown that either non-modification or incorrect modification of the precursor peptide significantly reduces or inhibits the biological activity of lanthipeptides [22,26].

## 3. Discussion

Actinobacterial genomes possess significant biosynthetic potential for producing antibacterial agents, accounting for over 50% of clinically available antibiotics [70,71,72]. Today, genome mining has become an invaluable tool for discovering new compounds in underexplored groups of actinobacteria, such as the genus *Embleya*. This phylum was reclassified in 2020 [44], resulting in numerous strains being added to public databases. Unfortunately, few studies have investigated novel natural products, such as emblestatin, an iron-chelating compound from *E. scabrispora* K20-0267 [73], and antibiotics chloptosins B and C from *Embleya* sp. MM621-AF10 [74]. To date, no studies have focused on genome mining or the discovery of a ribosomally synthesized and post-translationally modified peptide (RiPP) in this group.

Embleporicin represents the first class I lanthipeptide reported in the genus *Embleya*. This lanthipeptide was identified by genome mining in *Embleya* sp. NF3, an endophytic actinobacteria from *A. adstringens* [36]. Our bioinformatic analysis suggests that the embleporicin sequence shows homology among putative peptides of different species of *Embleya (*Figure 1). Potentially, these sequences are precursor peptides of class I lanthipeptides due to the presence of motif LDLD in the leader region, an important recognition site for biosynthetic enzymes [45,75]. Interestingly, homologous sequences were found in *Streptomyces* sp. SID3343 and *Actinoallomurus vinaceus* JCM 17939, we speculated that it is present in another genus. Likewise, the core region shares the same ring-forming amino acids serine and threonine, but to differentiate the other peptides, embleporicin harbors an additional cysteine residue and potentially contains one ring more than its homologous sequences. This characteristic would confer greater resistance and stability against heat and proteolytic degradation [3,68]. This hypothesis was verified with the expression assays, in which the samples were boiled to 85 °C, and embleporicin was resistant to the increase in temperature. 

Two regulator proteins are found within the embleporicin cluster. The MarR regulator is too far from the core biosynthetic pathway, while the TetR protein, EmpR, is adjacent to the cyclase sequence. We propose that the EmpR protein may play a role in embleporicin production, given its similarity to the AcrR1 regulator. Due to the protein’s likeness in ligand-binding sites, we cannot discount the possibility that EmpR is responsible for embleporicin synthesis. There may be another promoter sequence that has yet to be reported. However, a Gas Chromatography-Mass Spectrometry (GC-MS) analysis of extracts from *Embleya* sp. NF3 grown in ISP-2 medium did not identify any lanthipeptide-like structures after 29 days of incubation, but it is necessary to consider that this technique is not the right method for the lanthipeptides identification, so the production conditions of embleporicin in the host have not been elucidated [76]. 

We decided to utilize a CFPS system based on extracts from *E. coli*. This method has recently been applied to express bioactive peptides. Liu et al. [31] reported the production of nisin and its variants using CFPS for the first time. Similarly, Liu and colleagues [32] developed a system for producing antibacterial salivaricin variants, a class II lanthipeptide, resulting in the discovery of new antibacterial compounds. However, there have only been two studies on the CFPS of peptides derived from BGCs mined from genomic data of actinobacteria. The first study involved synthesizing the thiopeptide lactazole A from *Streptomyces lactacystinaeus* OM-6519 [77]. The second study detailed the production of a lasso peptide from *Thermobifida halotolerans* DSM 44931 [78]. These platforms provide a controllable environment that allows for precise manipulation and monitoring of substrates, intermediates, and products, facilitating the rapid identification of bottlenecks in metabolic pathways and resolving issues at specific stages [79]. Additionally, the versatile activity of post-translational modification enzymes enables the evaluation of various precursor peptide analogs and the testing of their antimicrobial activity without the need for purification [31,32]. In this context, the recent purification of nisin to a highly concentrated level from a CFPS platform using ammonium sulfate and electrodialysis confirms the system’s effectiveness in terms of purification [80]. In this work, we employed a low-cost CFPS system reported in 2022 [81]. The enzyme visualization through western blot analysis and the evaluation of peptide activity against *M. luteus* ATCC 9341, a model strain for assessing the antimicrobial potential of lanthipeptides, confirm the utility of this system for lanthipeptide production.

We observed that embleporicin exhibits an activity level like 90 UI of commercial nisin. Notably, embleporicin’s activity was only detectable with 600 μL of CFPS, indicating that the EmpA title is not very low, considering an approximate yield of 5 UI/mL of nisin in CFPS [31].

Additionally, the prediction server identified a protein from the CYP450 family, which may catalyze an oxidation reaction within the lanthipeptide, potentially adding a hydroxyl group. The presence of hydroxyl groups in certain lanthipeptides, such as cinnamycin [82], can enhance antibacterial activity. However, in variants of microbisporicin, this functional group has not been reported to modify antibacterial activity significantly [83]. Given the similarity of EmpO to MibO, we speculate that a similar effect may occur with embleporicin.

Furthermore, Liu et al. [31] reported that the NisB enzyme is a limiting factor in nisin production. We suspect a similar situation may arise with embleporicin production, where the EmpB enzyme’s expression was lower than the EmpC enzyme, as indicated by western blot analysis (Figure S4). Therefore, we anticipate higher activity when purified EmpB is introduced to CFPS.

On the other hand, embleporicin was only detectable in Tris-tricine gels and not in the western blot analysis. This challenge has also been noted in salivaricin B production, which required the addition of chaperone proteins to facilitate peptide solubilization [32]. There have been reports of using other antibodies, such as anti-leader peptide antibodies [84], for visualizing lanthipeptides.

Future research aims to enhance the efficiency of embleporicin production by identifying potential bottlenecks that may limit its synthesis. These include the amount of transcript generated and the stability and concentration of core biosynthetic enzymes. Additionally, techniques recently described [80] will be explored for embleporicin purification. Further analysis is also needed to detect lanthipeptide structures that could represent a new subclass of class I lanthipeptides from actinobacteria.

## 4. Materials and Methods

### 4.1. Genomic and Bioinformatic Analysis

The genome sequence of *Embleya* sp. NF3 was obtained from the National Center for Biotechnology Information (GenBank: MWQN00000000.1). It was screened using the antiSMASH v. 7.1.0 program [23], with the detection strictness set to “relaxed”, which allows the detection of well-defined clusters that contain all the necessary parts and partial clusters that are missing one or more functional parts and all additional features enabled. BAGEL4 [85] was utilized to confirm the results using the default settings, while the RiPPMiner [86] webserver was employed to estimate leader peptide cleavage.

Protein sequences were analyzed using InterPro [87] to identify the domains, functional sites, and families associated with each sequence (Table S3). Local alignments were conducted with BlastP from NCBI [88] to find conserved patterns (domains or motifs) with other protein sequences listed in the database. Concurrently, global multiple alignments were performed using the MUSCLE (Multiple Sequence Comparison by Log-Expectation) platform [89], and results were visualized with Jalview v2.11.4.1 [90]. The EMBOSS Needle webserver was used to calculate the percentage of identity, and UGENE software v51.0 [91] was applied to search for protein regulator recognition sequences. Additionally, a sequence logo was created using WebLogo v2.8.2 [92].

The structural models for EmpB, EmpC, and EmpR were constructed using AlphaFold [47] and visualized with ChimeraX-1.8 [93].

The SCO0268 sequences were obtained and downloaded from the Uniprot database [94]. Alignments were performed using the T-Coffee server [95], and these alignments were subsequently filtered with PhyML 3.0 [96] to reconstruct the phylogenetic tree using the Maximum Likelihood method based on the LG substitution model. A bootstrap analysis with 1000 resamplings was conducted. The phylogenetic tree was visualized and modified using the Interactive Tree Of Life v7 (iTOL) [97], with the NisA sequence as the outgroup.

### 4.2. Bacterial Strains and Media

*Escherichia coli* TOP10 (obtained from Invitrogen, C40406, Carlsbad, CA, USA) was used for biosynthetic gene cloning, *E. coli* JM109 (obtained from Promega, L2005, Madison WI, USA) for plasmids propagation, and *E. coli* BL21(DE)Star (obtained from Invitrogen, Carlsbad, CA, USA, C601003) for CFPS. LB medium (Luria-Bertani) contained 1% Tryptone, 0.5% yeast extract, and 0.5% sodium chloride and was used for strain propagation. For RNA isolation, *Embleya* sp. NF3 was cultivated on ISP-2 medium (0.4% yeast extract, 1.0% malt extract, 0.4% glucose). *M. luteus* ATCC 9341 (obtained from the American Type Culture Collection, Gaithersburg, MD, USA, and recently renamed as *Kocuria rhizophila*) was grown in TSB agar (1.7% Tryptone, 0.3% soy extract, 0.25% glucose 0.5% sodium chloride, 0.25% dipotassium phosphate, and 1.5% agar) for antimicrobial testing.

The vector pET22-b(+) was obtained from the collection laboratory, and pFGC_6XHis was donated by Dr. Guzmán-Chavez [81].

### 4.3. Clonation of empa, empB and empC Genes

The biosynthetic genes *empA*, *empB*, and *empC* were codon optimized for improved expression in *E. coli* and were synthesized within the pUCIDT and pJET1.2 cloning vectors by Integrated DNA Technologies (IDT) based in Coralville, IA, USA. The *empA* gene sequence includes a recognition site for the endoproteinase Furin between the leader peptide and the core peptide. Additionally, the *empB* and *empC* genes feature a histidine tag in the N-terminal region, along with *Bsa*I recognition sites in both the N-terminal and C-terminal regions.

The *empA, empB*, and *empC* genes were amplified using specific primer sequences via PCR (see Table S2) for subcloning into the expression vector. Following the manufacturer’s instructions, DNA amplification was performed using Q5 polymerase (M0492S, New England Biolabs, Ipswich, MA, USA, NEB^®^). The precursor peptide gene (*empA*) was cloned into the pET22-b(+) vector utilizing the restriction sites of *Nde*I and *Eco*RI enzymes (both from NEB^®^). The ligation was conducted using T4 DNA ligase (M1801, Promega, Madison, WI, USA).

The *empB* and *empC* genes were subcloned separately into the pFGC_6XHis vector using Golden Gate assembly. This process involved the purified insert, the receptor expression vector, 0.5 μL of *Bsa*I enzyme (R3733S, NEB^®^), 1.0 μL of T4 DNA ligase (Promega), 1.5 μL of the 10X buffer for T4 DNA ligase, and sterile Milli-Q water to bring the total volume to 15 μL. These constructs were then transformed into *E. coli* JM109 following the Froger and Hall protocol [98]. The results were confirmed by Sanger sequencing at Instituto de Fisiología Celular, UNAM.

The genetic constructs obtained were named pET22-*empA*, pFGC-*empB*, and pFGC-*empC*. The resulting expression products of these constructs were designated EmpA, EmpB, and EmpC. The precursor modified by the enzymes EmpB and EmpC was referred to as mEmpA. In contrast, the precursor modified by both EmpB and EmpC and cleaved by the endoproteinase Furin was named embleporicin.

### 4.4. Extraction of Total RNA from Embleya sp. NF3

Total RNA was extracted from *Embleya* sp. NF3 using a modified version of the Maes and Messens method [99]. Initially, *Embleya* sp. NF3 was grown for three days. After cultivation, the culture was centrifuged, and 200–300 mg of dried mycelium was weighed and ground in a mortar with 5 mL of Solution A (20 mM sodium acetate, 1 mM EDTA, pH 5.5). Next, 500 μL of 10% sodium dodecyl sulfate (SDS) and 5 mL of acid phenol were added to the extraction mixture.

The samples were agitated at 70 °C for 10 min, cooled immediately in a dry ice ethanol bath, and then centrifuged at 15,000× *g* for 10 min at 4 °C. The aqueous phase was extracted twice with an equal volume of acid phenol and chloroform. Following this, 0.1 volume of 3 M sodium acetate (pH 7.0) and 2.5 volume of absolute ethanol were added to the supernatant, which was then incubated overnight at −70 °C.

Finally, the samples were centrifuged at 15,000× *g* for 15 min at 4 °C. The pellet was washed twice with 70% ethanol and resuspended in 50 μL of Milli-Q water. This solution contains the total RNA.

### 4.5. Cell Extract Preparation and Expression of Genes of Interest by CFPS

The preparation of cell extracts from *E. coli* BL21 Star was conducted following the protocol established by Guzmán-Chávez et al. [81]. The genetic constructs pET22-*empA*, pFGC-*empB*, and pFGC-*empC* were expressed using cell-free protein synthesis (CFPS). Each reaction mix contained the three plasmids at a concentration of 20 nM, along with cell extract, 1X Wizard Buffer, total RNA from *Embleya* sp. NF3 (to dehydration reaction), polyethylene glycol 8000 (PEG-8000), and zinc chloride, as specified in Table 1. 

For the experiment, the recombinant plasmid pFGC_mScarlet [81] served as a positive control, which displayed a fluorescent red color in the reaction tube after the incubation period with a molecular weight of 26.4 kDa. The plasmid pFGC_6XHis was utilized as a negative control (Figure 3, Table S4). The reaction mixes were incubated for 14 h at 29 °C in 1.5 mL microtubes.

The samples were processed following the methods described by Liu et al. [31]. Briefly, each sample was warmed to 85 °C and maintained at this temperature for 5 min. After incubation, the sample was centrifuged for 1 min at 16,000× *g*. The supernatant was collected and incubated with Furin protease (P8077S, NEB^®^) at a ratio of 0.5 U per 25 μL of the supernatant. This mixture was then incubated at room temperature for 6 h. After this incubation, the leader peptide is expected to be cleaved from mEmpA, forming the mature peptide, i.e., embleporicin.

### 4.6. SDS-PAGE and Western-Blotting

The resolving gel used to visualize the bands corresponding to the molecular weights of the EmpB and EmpC enzymes was prepared with 0.75 mm SDS-PAGE gels containing 10% acrylamide/bisacrylamide, following the standard protocol [100]. The molecular weight marker, PageRuler™ Plus Prest Protein Ladder (26619), from Thermo Scientific™ (Waltham, MA, USA), was employed. The gel was placed in a room at 4 °C, and electrophoresis was conducted at a constant voltage of 70 V until the samples crossed the stacking gel, followed by an increase to 90 V for approximately 1.5 h. Coomassie Brilliant Blue G-250 (0.1%, 161-0400 Bio-Rad, Hercules, CA, USA) was used to stain the gel for 2 h. The gel was then destained overnight using a decolorizing solution made of 100 mL of acetic acid, 400 mL of methanol, and 500 mL of distilled water.

Western blotting was performed to confirm the expression of the EmpB and EmpC enzymes separated by Tris-glycine SDS-PAGE. Proteins were transferred to polyvinylidene fluoride (PVDF) membranes (Immobilon-P IPVH00010, Merck Millipore Ltd., Burlington, MA, USA) using a wet transfer system (Bio-Rad Mini Trans-Blot Cell Gel Electrophoresis System). The transfer buffer consisted of 25 mM Tris base, 0.19 M glycine, and 20% methanol, and the electrophoretic transfer was carried out at 90 V for 120 min at 4 °C. Membranes were blocked for 1 h at room temperature in Tris-buffered saline (TBS) with 0.05% Tween 20 (TBS-T) and 3% skimmed milk. Following this, membranes were incubated overnight with primary antibodies (IgG3 AB 6x-His, GeneTex [GT359] GTX628914) at 4 °C. After the primary antibody incubation, membranes were washed twice for 15 min in TBS-T. Membranes were then incubated for 2 h with a secondary antibody (ZyMax™ rabbit anti-mouse HPR, 81-6720 from Thermo Scientific™) at 4 °C, followed by two washes of 15 min in TBS-T. Bands were detected using the AP chromogen BCIP/NBT (GTX27468, GeneTex, Alton Pkwy Irvine, CA, USA).

### 4.7. Tricine SDS-PAGE

Tricine PAGE was used to visualize the embleporicin production, and reagents were prepared according to Haider et al. [101] with slight modifications. Briefly, we used acrylamide/Bis-acrylamide solution at 40% in proportion 29:1, and the electrophoresis was run at 130 V for 2 h.

Before the electrophoresis, each sample was warmed for 5 min at 85 °C after the incubation time. Subsequently, the sample was centrifuged for 1 min at 16,000× *g*, according to Liu et al. [29]. The supernatant was diluted at 50% with TBS buffer and mixed with 50% buffer sampling (2X: 100 mM Tris-HCl pH 6.8, 1% SDS, 4% 2-mercapthoethanol, 0.02% Coomassie Brilliant Blue G-250, 24% glycerol) and boiled 5 min before being loaded onto the gel. A SpectraTM Multicolor Low-Range Protein Ladder (26628) from Thermo Scientific™ was used. It was stained as Tris-Glycine SDS-PAGE. The western-blot analysis was conducted using a nitrocellulose membrane of 0.2 μm of pore (Amersham™ Protran™ 0.2 μm NC, 10600001, Sigma-Aldrich, San Luis, MO, USA) with the same conditions as Tris-glycine SDS-PAGE.

### 4.8. Antimicrobial Assay

The antimicrobial potential of embleporicin was tested against *M. luteus*. ATCC 9341. An overnight culture of *M. luteus* was inoculated onto Tryptic Soy Broth (TSB) plates, and 6 mm diameter wells were created in the agar. In one of the wells, 30 μL of concentrated embleporicin, derived from 600 μL of cell-free protein synthesis (CFPS) extract, was added. This extract was obtained through the vacuum concentration of 50 CFPS samples. We conducted the tests twice.

Negative control included CFPS with an empty vector concentrated in the same way as described above. This included CFPS with an empty vector (see Table S4) and an aliquot of Furin protease (NEB^®^). Additionally, different Nisin standards (Thermo Scientific™, 900 IU/mg,) were prepared in 0.02 M hydrochloric acid according to the method described by Tong et al. [102]. These Nisin standards served as positive control.

The plates were incubated at 4 °C for 1 h, followed by 18 h at 37 °C. After the incubation period, the inhibition halo was measured and recorded.

## 5. Conclusions

This study is the first to report the synthesis of a class I lanthipeptide derived from endophytic actinobacteria using cell-free protein synthesis (CFPS). We have named this peptide embleporicin. The expression of the core biosynthetic genes *empA*, *empB*, and *empC* produces a new peptide that exhibits antibacterial activity against *Micrococcus luteus*. However, its activity is only detectable at high concentrations of CFPS. In the future, we aim to enhance the efficiency of embleporicin production and evaluate its antimicrobial activity against multi-drug-resistant pathogenic strains, including both Gram-positive and Gram-negative bacteria. Our findings open up new opportunities to investigate the complex biosynthetic gene clusters (BGCs) of actinobacteria, explore the versatile functions of lanthipeptide enzymes, and promote the discovery of new ribosomally synthesized and post-translationally modified peptides (RiPPs) encoded in the genomes of rare actinobacteria such as *Embleya*. This research could lead to the development of novel pharmaceutical products.

## Figures and Tables

**Table 1:** Reaction mixture for immature embleporicin production.

Components	Concentration
*E. coli* BL21(Star) cell extract	4.0 μL
Buffer 4X Wizard	1X
pET22-*empA* (20 nM)	5 nM
pFGC-*empB* (20 nM)	
pFGC-*empC* (20 nM)	
Total RNA from *Embleya* sp. NF3	1.0 μL
40% PEG-8000	2%
ZnCl_2_ (300 μM)	10 μM
Milli-Q water	-
Final Volume	12.0 μL

## Data Availability

Data are available upon request.

## Supplementary Materials

The following supporting information can be downloaded at: https://www.mdpi.com/article/10.3390/antibiotics13121179/s1, Figure S1. Types of biosynthetic clusters identified in the genome of *Embleya* sp. NF3 using antiSMASH v. 7.1.0 program. Figure S2. Organization of the three clusters encoding putative class I lanthipeptides mined from *Embleya* sp. NF3 genome. Figure S3. Alignment of embleporicin post-translational modification enzyme sequences with other characterized sequences. Figure S4. Structural model of dehydratase enzyme EmpB (A), cyclase enzyme EmpC (B) and the regulator EmpR, when showing the ten α-helices. Figure S5. Visualization of EmpB and EmpC enzymes in Western-blot assays. Table S1. Functions of the genes within the embleporicin gene cluster. Table S2. Sequence of primers used for fragment amplification by PCR and sequencing. Table S3. Identifier from GenBank sequences used in this work. Table S4. Reaction mixture to produce precursor peptide EmpA, dehydratase enzyme EmpB, cyclase enzyme EmpC and modified EmpA.

## Abbreviations

The following abbreviations are used in this manuscript:

RiPPs	Ribosomally synthesized and post-translationally modified peptides
AMR	Microbial antibiotic resistance
CFPS	Cell-free protein synthesis
BGCs	Biosynthetic gene clusters
APC	Amino acid-polyamine carbocation permease
TBS-T	Tris-buffered saline with Tween
PVDF	Polyvinylidene difluoride
TSB	Tris-buffered saline
LanA	Lanthipeptide precursor peptide
LanB	Lanthipeptide dehydratase
LanC	Lanthipeptide cyclase
