# Supplementary material for: Embleporicin: A Novel Class I Lanthipeptide from the Actinobacteria Embleya sp. NF3
Source: Antibiotics (Basel). 2024 Dec 5;13(12):1179. doi: 10.3390/antibiotics13121179 (PMC11672506; doi:10.3390/antibiotics13121179)
Supplement: Supplementary file 1 [file antibiotics-13-01179-s001.zip › antibiotics-3333623-supplementary.pdf]

Supplementary Materials:

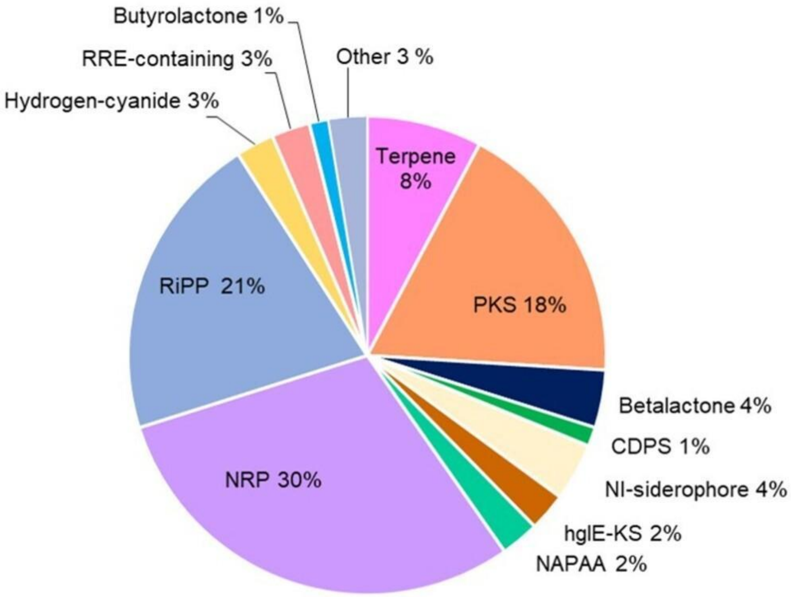

Figure S1. Types of biosynthetic clusters identified in the genome of *Embleya* sp. NF3 using antiSMASH v.7.1.0 program. The 30 % biosynthetically genes are associated with clusters that code for the production of nonribosomal peptides (NRP): 13 non-ribosomal peptides synthetase (NRPS), one NRP-metallophore and nine NRPS-like, the 21 % are associated with clusters that code for the production of ribosomally synthesized and post-translationally modified peptides (RiPPs): two RiPPs-like, eight lanthipeptides, three lassopeptides, one thiopeptide, one linaridine and one LAP (Linear azol(in)e-containing peptides.18 % clusters associated with the production of Polyketides synthase (PKS), 8 % with terpenes, 4 % with NI-siderophores, 4 % with betalactones, 3 % with RiPP recognition element (RRE-element containing cluster) 3 % with hydrogen-cyanide, 2 % with non-alpha poly-amino acids like e-Polylysin (NAPAA), 2 % with heterocyst glycolipid synthase-like PKS (hglE-KS), 1 % with butyrolactone, 1 % with tRNA-dependent cyclodipeptide synthases (CDPS) and 3 % with other clusters related to the production of secondary metabolites.

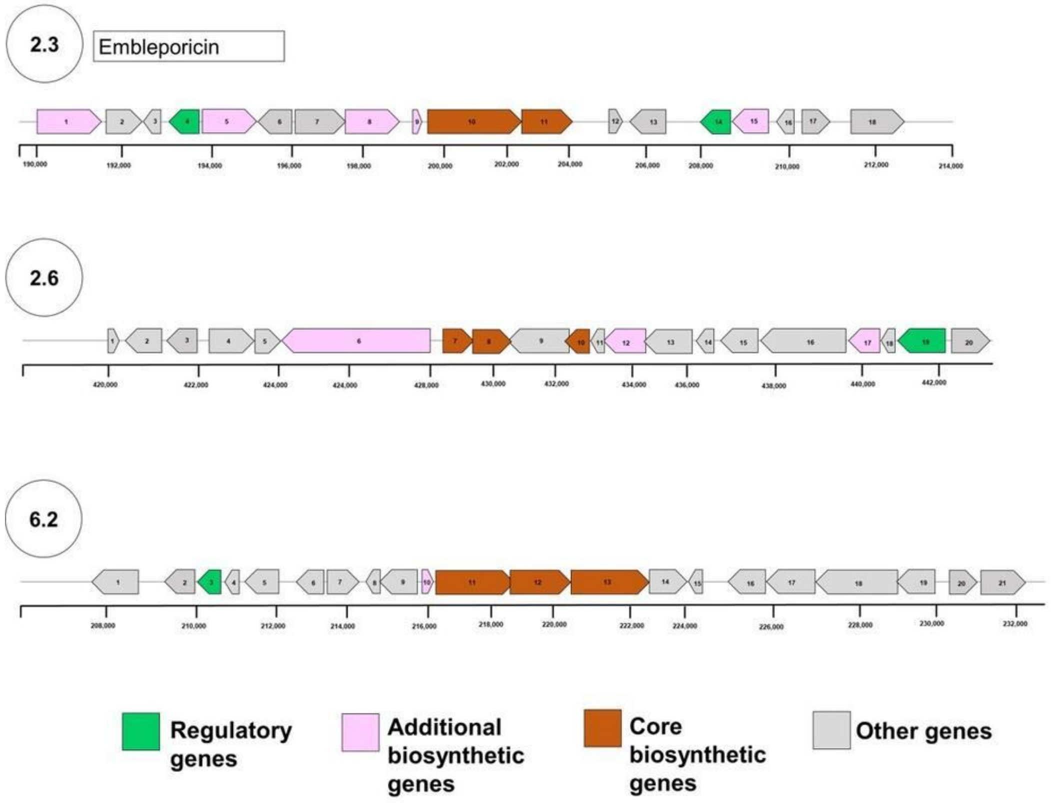

Figure S2. Organization of the three clusters encoding putative class I lanthipeptides mined from *Embleya* sp. NF3 genome. Results reported by antiSMASH v.7.1.0 program.

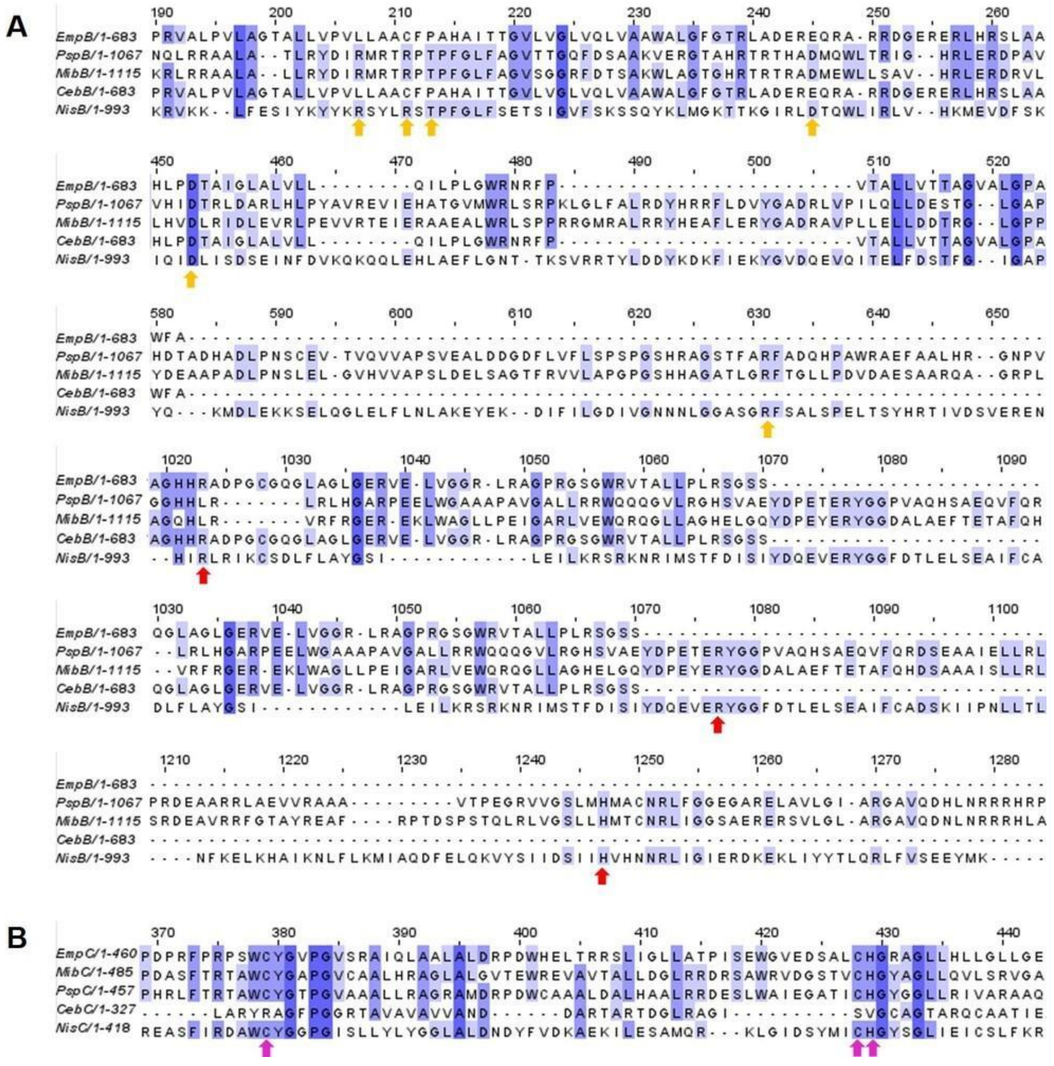

Figure S3. Alignment of embleporicin post-translational modification enzyme sequences with other characterized sequences. A) Sequence alignment of lantibiotic dehydratase, PspB (CCQ18696.1), MibB (ADK32555.1), CebB (QBK47605.1) and NisB (CAA48381.1). Only the region where amino acid residues reported as important for glutamylation (yellow arrows) and elimination (red arrows) are present is shown, according to a mutagenesis study performed on NisB [46]. B) Sequence alignment of lantibiotic cyclase, PspC (CCQ18697.1), MibC (ADK32556.1), CebC (QBK47608.1) and NisC (CAA48383.1). Only the region containing the amino acid residues involved in the catalytic triad of zinc ligand binding is highlighted (marked by a magenta arrow), based on previously reported findings [47].

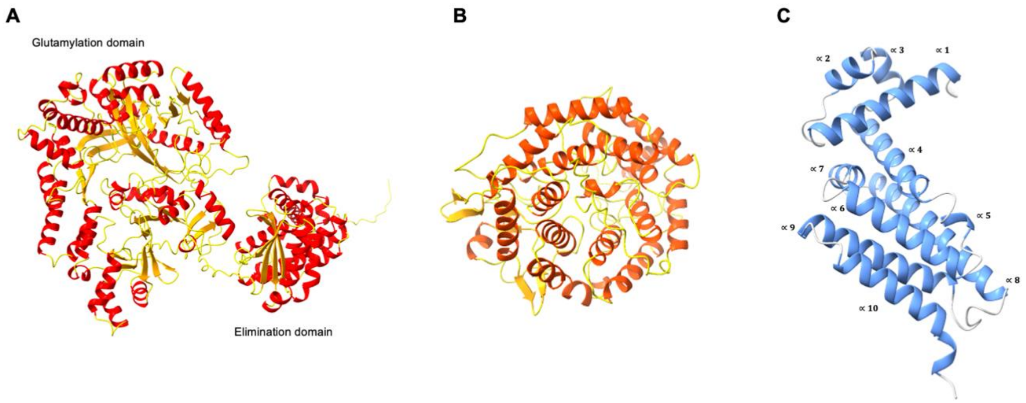

Figure S4. Structural model of dehydratase enzyme EmpB (A), cyclase enzyme EmpC (B) and the regulator EmpR, when showing the ten α-helices. Models were generated by AlphaFold [48] and visualized with ChimeraX-1.8 [94].

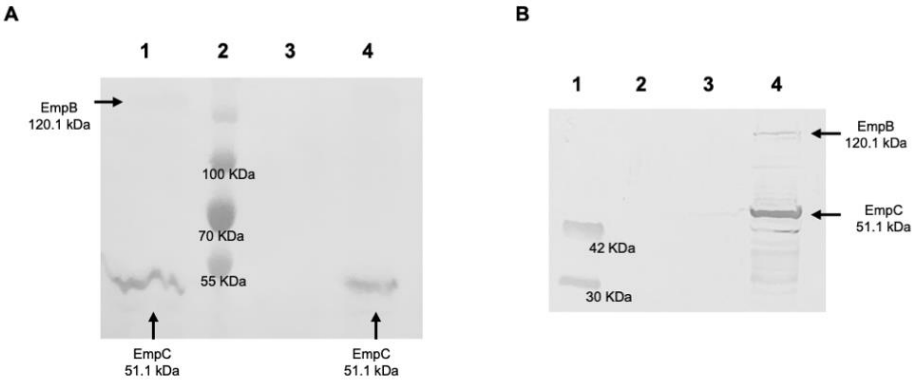

Figure S5. Visualization of EmpB and EmpC enzymes in Western-blot assays. (A) SDS-PAGE. Lane 1: Total CFPS with constructions pFGC_*empB* and pFGC_*empC*; Lane 2: PageRulerTM Plus Prestained Protein Ladder (26619); Lane 3: control negative (CFPS without plasmids); Lane 4: control negative (semi-purificated embleporicin). (B) Tricine SDS-PAGE. Lane 1: SpectraTM Multicolor Low Range Protein Ladder (26628); Lan 2 and 3: control negative (semi-purificated embleporicin). Lane 4: Total CFPS with constructions pFGC_*empB* and pFGC_*empC* and pET22-*empA*.

Table S1. Functions of the genes within the embleporicin gene cluster

**Gene**	**Identifier function**		**Length (amino acids)**	**Proposal function in biosynthesis embleporicin**
	**AntiSMASH V.7.0.1**	**BAGEL4**		

OPC78672.1	Aldehyde dehydrogenase PuuC	Not identifier	505	Not identifier
OPC78673.1	Peptidase C26	Not identifier	229	Not identifier
OPC78674.1	DUF4259 domain-containing protein	Not identifier	125	Not identifier
OPC79418.1	MarR family transcriptional regulator	Not identifier	190	Regulator protein
OPC78675.1	Amino acid permease	Uncharacterized amino acid permease	487	Regulator protein
OPC78676.1	Acetoacetate decarboxylase	Not identifier	277	Not identifier
OPC78677.1	Glutamine synthetase	Glutamine synthetase	452	Not identifier
OPC79419.1	Cytochrome CYP450	Methyl-branched lipid omega-hydroxylase	415	Oxidation
OPC78678.1	Precursor peptide	Precursor peptide	59	Precursor peptide
OPC79420.1	Lanthipeptide dehydratase	Lantibiotic dehydratase	1029	Dehydration
OPC78679.1	Lanthionine synthetase C family protein	Lantibiotic biosynthesis protein	460	Cyclation
OPC78680.1	Hypothetical protein	Not identifier	70	Not identifier
OPC78681.1	DNA-binding protein	DNA-binding protein HU2	232	Not identifier
OPC78682.1	TetR family transcriptional regulator	Uncheracterized HTH-type transcriptional regulator in *lacX* 3´region	200	Regulator protein

OPC78683.1	Alcohol dehydrogenase	Quinone oxidoreductase	320	Not identifier
OPC78684.1	DNA-binding protein	Not identifier	131	Not identifier
OPC78685.1	Gamma carbonic anhydrase family protein	Not identifier	175	Not identifier
OPC79421.1	Flavohemoprotein	Flavohemoprotei n	390	Not identifier

Table S2. Sequence of primers used for fragment amplification by PCR and sequencing

Primers	Sequence 5′-3′
M13 F	GTAAAACGACGGCCAGTG
M13 R	GGAAACAGCTATGACCATG
F*empB*	ttggtctcgAATGCGCCATTCCGAGCACGAGCACGGGCGCGGCGATG GCCCGCATGAAGGCTATGACGACGCCCGCGGAGATGAAGATACA TACCGTCCTGCCGGCCCCATCTTTGTTCGTATGGCTAGC
R*empB*	ttggtctcgaagcTCATCCATCATCGCGACGGTCACCTTCAACATCAGTA GGCCACTGGCGTGGAGGGTGTGCCAAGCGGCCAAGATGATCACT AGCGATGCCGCGTAAGATGGCATATCCACGACCCTCACTAG
F*emp*C	ttggtctcgAATGACTGACGAGCGCCGTACC
R*emp*C	ttggtctcgAAGCTCAAGCTACCATCAGGACACG
F1-*emp*B6XS	AGT CGC GTG GAC GTG GTA TTA
R1-*emp*6XS	GTA ACG GTC GGT TTC CGG TTC ATA GAC
F2-*emp*B6XS	AGT GGA CTT ACG CAT GGA CGC
R2-*emp*B6XS	CAT CTC ATG GCG AAG TAA CGT GCG

Lowercase letters represent nucleotides do not hybridize to the template and contain recognition site for *Bsa*I

Table S3. Identifier from GenBank sequences used in this work.

Number	Identifier	Productor organism
1	MWQN00000000.1	*Embleya* sp. NF3
2	SCO0268	*Streptomyces coelicolor* A3(2)

3	MET7300930.1	*Embleya* sp. NPDC005575
4	MEU0938789.1	*Embleya* sp. NPDC005971
5	WP_126642259.1	*Embleya hyalina*
6	WP_331771312.1	*Embleya* sp. NBC_00888
7	WP_331766507.1	*Embleya* sp. NBC_00896
8	WP_245599995.1	*Embleya scabrispora*
9	WP_237534230.1	*Streptomyces* sp. SID3343
10	WP_345438273.1	*Actinoallomurus vinaceus*
11	MYV96731.1	*Streptomyces* sp. SID3343
12	UPI001B334DB5	*Streptomyces* sp. GESEQ-13
13	UPI002F9179A4	*Embleya* sp. NBC_00896
14	A0AA96UI44	*Streptomyces* sp. Li-HN-5-13
15	UPI000B80C688	*Streptomyces* sp. F-7
16	UPI0011F2F4F8	*Streptomyces marokkonensis*
17	UPI001672F970	*Streptomyces viridiviolaceus*
18	UPI002886FC3D	*Streptomyces* sp. DSM 41529
19	UPI000C2CB6E6	*Kitasatospora fiedleri*
20	UPI002E15BE89	*Streptomyces* sp. NBC_01224
21	UPI002F912F03	*Embleya* sp. NBC_00888
22	UPI0031DB8EAA	*Streptomyces macrosporus*
23	A0A8I2J1Q6	*Streptomyces* sp. RM72
24	UPI0007C43EED	*Kitasatospora* sp. NRRL B-11411
25	UPI00234BAD64	*Streptomyces* sp. M92
26	UPI0024BF4E7D	*Streptomyces* sp. ML-6
27	UPI0028F31876	*Streptomyces tamarix*
28	UPI0031F0F751	*Kitasatospora albolonga*
29	BAA00602.1	*Lactococcus lactis*
30	CCQ18696.1	*Planomonospora alba*
31	ADK32555.1	*Microbispora corallina*
32	QBK47605.1	*Saccharopolyspora cebuensis*
33	CAA48381.1	*Lactococcus lactis*
34	CCQ18697.1	*Planomonospora alba*
35	ADK32556.1	*Microbispora corallina*
36	QBK47608.1	*Saccharopolyspora cebuensis*

37	CAA48383.1	*Lactococcus lactis*

Sequence of *Embleya* sp. NF3 genome (No. 1); sequences of SCO0268 family used in phylogenetic reconstruction (No. 2-29); sequences used in alignment of EmbB with other lantibiotic dehydratases (No. 30-33) and sequences used in alignment of EmbC with other lantibiotic cyclases (No. 34-37).

Table S4. Reaction mixture to produce precursor peptide EmpA, dehydratase enzyme EmpB, cyclase enzyme EmpC and modified EmpA.

**Components**	**Volume (μl)**					
	**Positive control**	**EmpA**	**EmpB**	**EmpC**	**mEmpA**	**Negative control**
E. coli BL21(Star) cell extract	4.0	4.0	4.0	4.0	4.0	4.0
Buffer 4X Wizard	3.0	3.0	3.0	3.0	3.0	3.0
pFGC_mScarlet* (20 nM)	3.0	-	-	-	-	-
pET22_*empA* (20 nM)	-	3.0	-	-	1.0	-
pFGC_*empB* (20 nM)	-	-	3.0	-	1.0	-
pFGC_*empC* (20 nM)	-	-	-	3.0	1.0	-
pFGC_6XHis+ (20nM)	-	-	-	-	-	3.0
RNA from *Embleya* sp. NF3 (1500-2000 ng)	-	-	-	-	1.0	1.0
40% PEG-8000	0.6	0.6	0.6	0.6	0.6	0.6
$ZnCl_2$ (100 μM)	1.2	1.2	1.2	1.2	-	-
$ZnCl_2$ (300 μM)	-	-	-	-	0.4	0.4
Milli-Q water	0.2	0.2	0.2	0.2	0	0
**Final Volume**	12.0 μL					

*The construct pFGC_mScarlet encoding the mScarlet fluorescent protein, was used as a positive control. Its production was monitored by observing the colour change in the in vitro reaction after the incubation period. +The empty plasmids pFGC_6XHis was used as a negative control.
